# Limited Health Knowledge as a Reason for Non-Use of Four Common Complementary Health Practices

**DOI:** 10.1371/journal.pone.0129336

**Published:** 2015-06-17

**Authors:** Adam Burke, Richard L. Nahin, Barbara J. Stussman

**Affiliations:** 1 Institute for Holistic Health Studies, Department of Health Education, San Francisco State University, San Francisco, California, United States of America; 2 National Center for Complementary and Integrative Health, National Institutes of Health, Bethesda, Maryland, United States of America; Texas Tech University Health Science Centers, UNITED STATES

## Abstract

**Background:**

Complementary health practices are an important element of health/healthcare seeking behavior among adults in the United States. Reasons for use include medical need, prevention and wellness promotion, and cultural relevance. Survey studies published over the past several decades have provided important information on the use of complementary health practices, such as acupuncture and yoga. A review of the literature, however, reveals an absence of studies looking specifically at who does not use these approaches, and why not.

**Methods:**

To explore this issue two samples were created using data from the 2007 National Health Interview Survey Complementary and Alternative Medicine supplement. Of particular interest was the relationship between lack of health knowledge, as a reason for non-use, and key independent variables. The first sample was comprised of individuals who had never used any of four common complementary health practices -- acupuncture, chiropractic, natural products, and yoga. The second was a subset of those same non-users who had also reported low back pain, the most frequently cited health concern related to use of complementary therapies.

**Results:**

A hypothesized association between lack of health knowledge, lower educational attainment, and other key socioeconomic indicators was supported in the findings. Although it was hypothesized that low back pain would be associated with greater information seeking, regardless of level of education, that hypothesis was not supported.

**Conclusion:**

Lack of knowledge was found to affect utilization of common complementary health practices, regardless of the potentially motivating presence of back pain. Disparities in the utilization of complementary medicine, related to educational attainment and other socioeconomic factors, may negatively affect quality of care for many Americans. Creative approaches are needed to help reduce inequities in understanding and improve access to care for underserved populations.

## Introduction

Complementary health practices (CHP), such as acupuncture and yoga, are important elements of health/healthcare seeking behavior among adults in the United States. Reasons for use include medical need, prevention and wellness promotion, and cultural relevance [1]. In terms of medical need, CHP use has been found to be associated with having one or more medical conditions, with having a diagnosed chronic disease, such as low back pain, and with having been hospitalized during the past year [2–4]. In addition to medical need, numerous studies have also found a relationship between use of CHP's and the practice of other conventional preventive health behaviors. CHP users have been shown to be more likely to engage in leisure time physical activity, to be former smokers, to consume alcohol moderately, have a healthier body mass index, eat a lower fat diet, and utilize preventive medical services, such as cholesterol screening [5–8]. Other important insights into the characteristics of individuals who use CHP's include being more prevalent among women, individuals aged 30–69, living in the West, having higher levels of education, and not being poor [2, 3, 9].

Despite all of this research, a review of the literature reveals an absence of studies looking specifically at who does not use CHP’s and why not. With rare exception, such as one study looking specifically at non-use of acupuncture [10], there have been virtually no detailed analyses of reasons for non-use. To explore this issue a retrospective cross-sectional analysis of the 2007 National Health Interview Survey (NHIS) Complementary and Alternative Medicine Supplement was performed. Of particular interest was respondent self-reported lack of knowledge (of four common complementary practices) as a reason for non-use, suggesting lower health knowledge and health literacy.

Health literacy has been defined as, “The degree to which individuals have the capacity to obtain, process, and understand basic information and services needed to make appropriate decisions regarding their health" [11]. Conceptual knowledge of health and healthcare is an important element of health literacy, such as knowledge of treatment options [11–12]. Limited health knowledge and health literacy can significantly affect utilization of conventional healthcare services, the practice of preventive health behaviors, disease management, disease outcomes, and healthcare expenditures [13–17]. Correlates of limited health knowledge included lower levels of functional health literacy [18–19], lower educational attainment [20], lower socioeconomic status, and race [21–23].

Research provides information on the relationship between health knowledge and conventional healthcare utilization. Corresponding information on the relationship between health knowledge and use of complementary healthcare practices, however, is missing. To explore this issue, lack of knowledge of four commonly used complementary health practices was examined, as a reason for their non-use. A hypothesized association between lack of knowledge, lower educational attainment, and other key socioeconomic indicators was supported in the findings. An additional analysis was conducted with a subset of those same non-users who also reported low back pain. Low back pain was chosen as the main health complaint for this analysis for several important reasons. First, back pain is the most prevalent health condition related to use of complementary therapies [2–3]. Research also suggests that individuals with low back pain actively seek out information and treatment options for their condition [24], which would presumably include information on complementary health practices. Finally, best practice clinical guidelines for the management of back pain include several complementary health practices [25], and Medicare provides coverage for chiropractic treatment of back pain [26]. For these reasons it was hypothesized that this subset of respondents with back pain would be more likely to seek information and possess knowledge of CHP options for back pain, regardless of level of educational attainment. This anticipated outcome was not supported in the findings. Taken together these results suggest that factors such as lower educational attainment contribute to disparities in access to potentially useful therapeutic services by virtue of limited health knowledge, even among those with back pain.

## Materials and Methods

### Data Source

The National Health Interview Survey (NHIS) is an annual survey of the health of the United States civilian, non-institutionalized population. It is conducted by the National Center for Health Statistics, Centers for Disease Control and Prevention (CDC). It uses an in-person, computer-assisted interviewing method of administration. The survey contains four main modules: Household, Family, Sample Child, and Sample Adult. The first two modules collect health and socio-demographic information on each member of all families residing within a sampled household. Within each family, additional information is collected from one randomly selected adult (the “sample adult”) aged 18 years or over. The survey uses a multi-stage clustered sample design, and oversamples black, Asian and Hispanic populations to allow for more precise estimation of health characteristics in these growing minority populations. The 2007 NHIS was approved by the National Center for Health Statistics Research Ethics Review Board on October 18, 2006. Verbal consent was allowed by the NCHS Ethics Review Board and was obtained by Census Bureau interviewers from all survey respondents prior to the start of the interview. Public use data files are available online (http://www.cdc.gov/NCHS/nhis/nhis_2007_data_release.htm).

### Population of Study

The study was based on the 2007 National Health Interview Survey. The core dataset consisted of 75,764 individuals from 29,915 families in 29,266 households. The total household response rate was 87.1%. From the participating households, 23,393 adults completed interviews, resulting in an overall sample adult response rate of 67.8%. Administration of the 2007 NHIS included a 15-minute Complementary and Alternative Medicine supplement. The supplement contained questions on 36 complementary approaches used in the United States, including 10 provider-based approaches, such as acupuncture, chiropractic/osteopathic manipulation (chiropractic), and traditional healers, and 26 self-care approaches, such as special diets, yoga, and use of non-vitamin/non-mineral natural products (natural products, such as botanical dietary supplements). Similar complementary and alternative medicine NHIS supplement surveys were also conducted in 2002 and 2012.

Unique to the 2007 NHIS supplement, additional information on reasons for non-use was collected whenever respondents indicated they had not used any of the five most common complementary health practices—acupuncture, chiropractic, natural products, yoga, and meditation. Four of these practices were selected for the current analysis: acupuncture, chiropractic, natural products, and yoga. These approaches were selected because each one represents one of the four major domains used to categorize the 36 complementary health practices examined in the 2007 NHIS Supplement: (1) alternative medical systems (acupuncture), (2) biologically-based therapies (natural products), (3) manipulative and body-based therapies (chiropractic), and (4) mind-body therapies (yoga). Also, two were provider-based (acupuncture and chiropractic) and two were self-care oriented (yoga and natural products), providing a balanced representation of both types.

From the core NHIS dataset two samples were created for analysis. The first was a sample of individuals who had never used acupuncture, chiropractic, natural products, or yoga. This resulted in a study population of 13,128 individuals, or 55% (SE 0.52) of all interviewed sample adults. Analyses presented in Table 1a–1c and S1 Table are based on this sample. A second subset was created from this non-user sample, of respondents who had also reported low back pain in the previous 3 months (n = 2,580).

**Table 1a-c:** Ten reasons for non-use of four common complementary health practices by population samples analyzed.

Reason for Non-Use*	Overall		Acupuncture		Chiropractic		Natural Products		Yoga	
	N**	% (SE)	N	% (SE)	N	% (SE)	N	% (SE)	N	% (SE)
**1a. Full Sample**										
1. No reason	69,011	56.1 (0.81)	39,225	31.9 (0.69)	38,353	31.2 (0.68)	46,392	37.7 (0.75)	47,083	38.3 (0.74)
2. Do not need it	52,379	42.6 (0.84)	29,901	24.3 (0.63)	38,473	31.3 (0.80)	29,967	24.3 (0.64)	22,165	18.0 (0.62)
3. Never thought about	48,497	39.4 (0.70)	27,529	22.4 (0.59)	21,831	17.7 (0.60)	26,120	21.2 (0.60)	29,841	24.2 (0.65)
4. Never heard	28,998	23.6 (0.70)	18,959	15.4 (0.56)	16,656	13.5 (0.59)	13,440	10.9 (0.52)	15,085	12.3 (0.54)
5. Do not believe	13,677	11.1 (0.44)	5,947	4.8 90.29)	5,724	4.7 (0.27)	6,255	5.1 (0.27)	3,825	3.1 (0.20)
6. Some other reason	9,620	7.8 (0.36)	3,374	2.7 (0.19)	1,622	1.3 (0.14)	1,997	1.6 (0.15)	6,084	4.9 (0.31)
7. It costs too much	3,717	3.0 (0.21)	1,415	1.1 (0.12)	2,021	1.6 (0.15)	1,240	1.0 (0.10)	787	0.6 (0.09)
8. It is not safe to use	1,424	1.2 (0.12)	411	0.3 (0.07)	561	0.5 (0.07)	504	0.4 (0.06)	164	0.1 (0.04)
9. No evidence it works	1,094	0.9 (0.10)	475	0.4 (0.06)	289	0.2 (0.05)	540	0.4 (0.07)	98	0.1 (0.02)
10. Provider said no	737	0.6 (0.10)	123	0.1 (0.03)	358	0.3 (0.07)	307	0.2 (0.06)	21	0.0 (0.01)
**1b. Back Pain Only**										
1. No reason	13,482	57.7 (1.28)	7,567	32.4 (1.21)	7,343	31.4 (1.21)	9,001	38.5 (1.24)	8,987	38.5 (1.34)
2. Do not need it	8,403	36.0 (1.26)	4,223	18.1 (1.12)	5,358	22.9 (1.15)	4,361	18.7 (0.99)	3,348	14.3 (0.91)
3. Never thought about	9,408	40.3 (1.26)	5,236	22.4 (1.01)	4,223	18.1 (0.92)	4,962	21.2 (1.02)	5,366	23.0 (1.09)
4. Never heard	6,169	26.4 (1.15)	3,831	16.4 (0.96)	3,409	14.6 (0.94)	2,673	11.4 (0.81)	2,985	12.8 (0.82)
5. Do not believe	3,424	14.7 (0.98)	1,325	5.7 (0.61)	1,537	6.6 (0.69)	1,623	6.9 (0.64)	1,074	4.6 (0.57)
6. Some other reason	2,719	11.6 (0.88)	1,072	4.6 (0.56)	633	2.7 (0.42)	690	3.0 (0.55)	1,641	7.0 (0.79)
7. It costs too much	1,443	6.2 (0.61)	605	2.6 (0.39)	925	4.0 (0.49)	410	1.8 (0.27)	293	1.3 (0.25)
8. It is not safe to use	514	2.2 (0.36)	98	0.4 (0.13)	295	1.3 (0.28)	145	0.6 (0.16)	77	0.3 (0.14)
9. No evidence it works	266	1.1 (0.28)	101	0.4 (0.19)	55	0.2 (0.12)	82	0.4 (0.12)	47	0.2 (0.11)
10. Provider said no	272	1.2 (0.24)	29	0.1 (0.07)	138	0.6 (0.17)	128	0.5 (0.17)	40	0.0 (0.02)
**1c. No Back Pain**										
1. No reason	55,476	55.7 (0.88)	31,639	31.8 (0.74)	30,994	31.1 (0.75)	37,342	37.5 (0.80)	38,067	38.2 (0.79)
2. Do not need it	43,956	44.1 (0.93)	25,664	25.8 (0.71)	33,096	33.2 (0.89)	25,604	25.7 (0.70)	18,816	18.9 (0.69)
3. Never thought about	38,999	39.2 (0.79)	22,234	22.3 (0.66)	17,526	17.6 (0.66)	21,109	21.2 (0.65)	24,398	24.5 (0.72)
4. Never heard	22,829	22.9 (0.72)	15,129	15.2 (0.59)	13,248	13.3 (0.63)	10,767	10.8 (0.54)	12,100	12.1 (0.57)
5. Do not believe	10,242	10.3 (0.47)	4,612	4.6 (0.30)	4,187	4.2 (0.30)	4,622	4.6 (0.30)	2,750	2.8 (0.21)
6. Some other reason	6,878	6.9 (0.38)	2,290	2.3 (0.22)	989	1.0 (0.14)	1,307	1.3 (0.13)	4,432	4.4 (0.30)
7. It costs too much	2,273	2.3 (0.20)	810	0.8 (0.11)	1,096	1.1 (0.14)	830	0.8 (0.11)	494	0.5 (0.08)
8. It is not safe to use	909	0.9 (0.13)	313	0.3 (0.09)	266	0.3 (0.06)	359	0.4 (0.07)	87	0.1 (0.04)
9. No evidence it works	822	0.8 (0.19)	368	0.4 (0.07)	227	0.2 (0.05)	451	0.5 (0.08)	45	0.0 (0.01)
10. Provider said no	465	0.5 (0.10)	94	0.1 (0.03)	220	0.2 (0.08)	108	0.2 (0.06)	17	0.0 (0.01)

*List of reasons for non-use given to participants

1. No reason

2. Don’t need it

3. Never thought about it

4. Never heard of it / don’t know much about it

5. Don’t believe in it / it doesn’t work

6. Some other reason

7. It costs too much

8. It is not safe to use

9. Medical science has not shown that it works

10. A health care provider told me not to use it

**Population estimates, numbers in thousands

Several variables used in the regression analyses contained missing data, thereby reducing the sample size of each unique model based on the data available for the variables included. Sample sizes are shown on the column heading of Tables 2–5 for each unique model. The lowest sample sizes were seen for models that contained poverty status and/or body-mass index variables, since these variables had the highest number of missing observations. Models that contained both of them would have lost 2,469 observations due to missing data. In order to ensure the models had comparable demographic distributions, we examined the frequencies for each variable shown in S1 Table for each unique model and found little variation across models. The only statistically significant differences found were for 'lifetime abstainer from alcohol' (do not need acupuncture, and never heard of acupuncture, chiropractic, and yoga, approximately 10% lower than the full sample) and '65+ age group' (never heard of chiropractic and yoga were approximately 15% lower than the full sample).

**Table 2:** Adjusted odds ratios and 99% confidence interval estimates of items independently associated with choosing 'lack of knowledge' as a reason for non-use of four complementary health practices, full sample.

		Four Common Complementary Health Practices											
		Chiropractic			Acupuncture			Natural Products			Yoga		
		(n = 10,580)			(n = 10,591)			(n = 12,365)			(n = 10,578)		
		AOR	99% CI	P value	AOR	99% CI	P value	AOR	99% CI	P value	AOR	99% CI	P value
**DEMOeGRAPHICS**													
Sex	Female (ref = male)				0.87	.73–1.03	0.034				0.87	.72–1.04	0.049
Age	18–24				1		0.009	1		0.139			
	25–44				0.76	.57–1.02		0.73	.5–1.07				
	45–64				0.67	.49-.91		0.73	.5–1.07				
	65+				0.8	.59–1.09		0.81	.54–1.22				
Hispanic	Yes (ref = no)				1.25	.95–1.64	0.037						
Region	South				1		0.002				1		0.044
	Northeast				0.86	.62–1.18					0.73	.5–1.06	
	Midwest				0.93	.7–1.24					0.84	.6–1.16	
	West				0.59	.41-.84					0.71	.48–1.04	
Poverty	Poor	1		<0.0001	1		<0.0001				1		0.004
	Near poor	0.95	.72–1.26		0.97	.75–1.27					0.97	.7–1.33	
	Not poor	0.63	.46-.86		0.61	.45-.82					0.72	.51–1.02	
Education	<High school	1		<0.0001	1		<0.0001	1		<0.0001	1		<0.0001
	High school	0.71	.57-.9		0.66	.53-.82		0.57	.46-.71		0.62	.49-.78	
	Some college or >	0.54	.41-.72		0.42	.33-.55		0.49	.39-.62		0.44	.33-.58	
**HEALTH STATUS**													
Functional limitations	Yes (ref = no)	0.78	.62-.98	0.005									
Back pain	Yes (ref = no)	1.11	.87–1.42	0.276	0.99	.80–1.22	0.865	0.99	.81–1.22	0.915	0.95	.76–1.19	0.586
**HEALTH BEHAVIORS**													
Activity levels	Never	1		<0.0001	1		<0.0001	1		0.0002	1		<0.0001
	Some	0.71	.54-.92		0.79	.63-.99		0.76	.59-.98		0.72	.56-.92	0.01
	Regular	0.6	.46-.77		0.67	.53-.85		0.67	.51-.89		0.65	.5-.83	
Alcohol consumption	Lifetime abstainer	1		<0.0001	1		0.0001	1		0.082	1		<0.0001
	Former drinker	0.66	.49-.87		0.75	.57-.99		0.8	.6–1.05		0.74	.56-.97	
	Current infrequent	0.72	.56-.92		0.83	.66–1.05		0.91	.7–1.18		0.72	.56-.94	
	Current moderate/ Heavy	0.5	.37-.67		0.59	.44-.8		0.77	.56–1.06		0.53	.37-.77	
**HEALTHCARE ACCESS**													
Not afford ancillary care	Yes (ref = no)	1.13	.86–1.48	0.253	1.23	.98–1.54	0.021	1.14	.89–1.47	0.167	1.29	1.01–1.64	0.008
Delayed care, non-cost	Yes (ref = no)	1.08	.8–1.46	0.507				1.22	.88–1.68	0.115			

**Table 3:** Adjusted odds ratios and 99% confidence interval estimates of items independently associated with choosing 'lack of need' as a reason for non-use of four complementary health practices, full sample.

		Four Common Complementary Health Practices											
		Chiropractic			Acupuncture			Natural Products			Yoga		
		(n = 12,341)			(n = 10,779)			(n = 12,657)			(n = 13012)		
		AOR	99% CI	p value	AOR	99% CI	p value	AOR	99% CI	p value	AOR	99% CI	p value
**DEMOGRAPHICS**													
Sex	Female (ref = male)	1.08	.94–1.23	0.162							0.69	.6-.81	<0.0001
Hispanic	Yes (ref = no)	1.21	1.01–1.43	0.006				1.38	1.13–1.68	<0.0001			
Race	White				1		0.027						
	Black				0.86	.69–1.06							
	Other				1.16	.93–1.44							
Region	South	1		0.0001							1		0.0001
	Northeast	0.66	.5-.86								0.73	.55-.99	
	Midwest	1.16	.86–1.57								1.17	.83–1.64	
	West	1.02	.83–1.24								1.24	.98–1.58	
Poverty	Poor				1		0.0008						
	Near poor				1.02	.77–1.35							
	Not poor				1.31	1.02–1.68							
Education	<High school	1		0.021	1		0.004	1		0.033			
	High school	1.17	.97–1.42		1.08	.87–1.35		1.09	.91–1.31				
	Some college or >	1.22	1.01–1.49		1.26	1.04–1.54		1.21	1–1.45				
**HEALTH STATUS**													
Self report health status	Excel/VG/Good	1		0.332	1		0.209	1		0.573			
	Fair/Poor	0.92	.73–1.15		0.88	.69–1.14	0.05	0.95	.74–1.22				
Functional limitations	Yes (ref = no)							0.89	.76–1.05	0.078	0.86	.7–1.07	0.075
ER past 12 months	Yes (ref = no)	0.83	.7-.99	0.005	0.78	.64-.95	0.001	0.83	.68–1.0	0.01	0.79	.64-.97	0.004
Back Pain	Yes (ref = no)	0.65	.53-.78	<0.0001	0.74	.58-.93	0.0009	0.77	.64-.92	0.0003	0.8	.65-.98	0.006
**HEALTH BEHAVIORS**													
Activity levels	Never	1		<0.0001	1		<0.0001	1		0.001			
	Some	1.64	1.38–1.94		1.37	1.14–1.66		1.26	1.05–1.52				
	Regular	1.47	1.23–1.77		1.37	1.13–1.66		1.24	1.03–1.48				
Alcohol consumption	Lifetime abstainer	1		0.002									
	Former drinker	1.31	1.05–1.63										
	Current infrequent	1.22	1.04–1.43										
	Current moderate/ Heavy	1.32	1.04–1.67										
**HEALTHCARE ACCESS**													
Usual place of care	No (ref = yes)	1.25	1.01–1.55	0.008	1.15	.94–1.42	0.076	1.15	.95–1.4	0.078			
Not afford ancillary care	Yes (ref = no)	0.82	.67–1	0.01	0.88	.7–1.11	0.144				0.78	.63-.98	0.006
Delayed care, non-cost	Yes (ref = no)							0.71	.56-.9	0.0002	1.13	.83-.154	0.314
Health insurance	Private	1		0.095									
	Public	0.94	.78–1.12										
	None	0.85	.69–1.04										

**Table 4:** Adjusted odds ratios and 99% confidence interval estimates of items independently associated with choosing 'lack of knowledge' as a reason for non-use of four complementary health practices, back pain only sample.

		Four Common Complementary Health Practices											
		Chiropractic			Acupuncture			Natural Products			Yoga		
		(n = 2,123)			(n = 2,123)			(n = 2,455)			(n = 2,119)		
		AOR	99% CI	p value	AOR	99% CI	p value	AOR	99% CI	p value	AOR	99% CI	p value
**DEMOGRAPHICS**													
Sex	Female (ref = male)				0.96	.66–1.4	0.778				1.19	.76–1.84	0.32
Age	18–24				1		0.481	1		0.534			
	25–44				1.21	.61–2.4		1.18	.56–2.50				
	45–64				0.97	.49–1.93		1.41	.7–2.82				
	65+				1.26	.62–2.57		1.2	.54–2.68				
Hispanic	Yes (ref = no)				1.08	.63–1.84	0.702						
Region	South				1		0.214				1		0.013
	Northeast				0.7	.41–1.2					0.43	.23-.84	
	Midwest				0.94	.6–1.48					0.83	.5–1.38	
	West				0.67	.35–1.29					0.78	.43–1.43	
Poverty	Poor	1		0.534	1		0.028				1		0.796
	Near poor	1.09	.68–1.75		1.25	.76–2.05					1.07	.63–1.81	
	Not poor	0.89	.57–1.41		0.8	.49–1.31					1.15	.66–2.0	
Education	<High school	1		0.055	1		0.254	1		<.0001	1		0.001
	High school	0.71	.43–1.17		1.28	.56–1.9		0.46	.28-.75		0.66	.4–1.08	
	Some college or >	0.63	.38–1.04		1.1	.72–1.68		0.5	.31-.8		0.5	.31-.82	
**HEALTH STATUS**													
Functional limitations	Yes (ref = no)	1.01	.64–1.6	0.96									
**HEALTH BEHAVIORS**													
Activity levels	Never	1		0.001	1		0.0003	1		0.044	1		0.0001
	Some	0.73	.43–1.23		0.65	.4–1.04		0.71	.43–1.18		0.62	.37–1.03	
	Regular	0.39	.2-.75		0.4	.21-.75		0.59	.32–1.1		0.32	.15-.66	
Alcohol consumption	Lifetime abstainer	1		0.03	1		0.43	1		0.834	1		0.025
	Former drinker	0.63	.37–1.07		0.76	.47–1.25		0.99	.61–1.62		0.78	.47–1.28	
	Current infrequent	0.64	.4–1.05		0.85	.51–1.4		0.84	.49–1.45		0.56	.34-.92	
	Current moderate/ Heavy	0.66	.35–1.27		0.76	.42–1.37		0.91	.5–1.63		0.67	.33–1.35	
**HEALTHCARE ACCESS**													
Not afford ancillary care	Yes (ref = no)	1.24	.77–2.01	0.245	1.38	.91–2.09	0.048	1.51	.98–2.32	0.0145	1.6	1.06–2.41	0.003
Delayed care, non-cost	Yes (ref = no)	1.07	.64–1.81	0.725				1.21	.7–2.08	0.367			

**Table 5:** Adjusted odds ratios and 99% confidence interval estimates of items independently associated with choosing 'lack of need' as a reason for non-use of four complementary health practices, back pain only sample.

		Four Common Complementary Health Practices											
		Chiropractic			Acupuncture			Natural Products			Yoga		
		(n = 2,455)			(n = 2,149)			(n = 2,498)			(n = 2,556)		
		AOR	99% CI	p value	AOR	99% CI	p value	AOR	99% CI	p value	AOR	99% CI	p value
**DEMOGRAPHICS**													
Sex	Female (ref = male)	0.95	.69–1.31	0.663							0.57	.39-.85	0.0003
Hispanic	Yes (ref = no)	0.98	.64–1.5	0.92				1.25	.83–1.87	0.169			
Race	White				1		0.863						
	Black				0.91	.56–1.48							
	Other				0.93	.53–1.64							
Region	South	1		0.189							1		0.457
	Northeast	0.72	.42–1.25								0.76	.40–1.42	
	Midwest	0.71	.44–1.13								0.99	.63–1.58	
	West	0.85	.55–1.32								0.78	.46–1.32	
Poverty	Poor				1		0.156						
	Near poor				0.8	.43–1.49							
	Not poor				1.17	.71–1.92							
Education	<High school	1		0.254	1		0.366	1		0.294			
	High school	1.28	.56–1.9		1.08	.66–1.79		1.23	.82–1.85				
	Some college or >	1.1	.72–1.68		1.28	.8–2.06		1.26	.84–1.89				
**HEALTH STATUS**													
Self report health status	Excel/VG/Good	1		0.222	1		0.053	1		0.382			
	Fair/Poor	0.84	.57–1.22		0.71	.46–1.12		1.16	.75–1.8				
Functional limitations	Yes (ref = no)							0.75	.54–1.03	0.02	0.79	.54–1.17	0.124
ER past 12 months	Yes (ref = no)	0.83	.58–1.18	0.171	0.69	.45–1.04	0.02	0.96	.67–1.38	0.782	0.72	.45–1.13	0.06
**HEALTH BEHAVIORS**													
Activity levels	Never	1		0.008	1		0.161	1		0.764			
	Some	1.55	1.08–2.25		1.27	.78–2.07		1.13	.74–1.72				
	Regular	1.35	.86–2.1		0.85	.5–1.42		1.06	.65–1.75				
Alcohol consumption	Lifetime abstainer	1		0.371									
	Former drinker	1.04	.64–1.68										
	Current infrequent	1.25	.82–1.91										
	Current moderate/Heavy	0.99	.54–1.78										
**HEALTHCARE ACCESS**													
Usual place of care	No (ref = yes)	1.12	.71–1.78	0.519	1.29	.78–2.14	0.184	1.56	1.06–2.28	0.003			
Not afford ancillary care	Yes (ref = no)	0.81	.54–1.21	0.169	1.01	.66–1.55	0.947				0.93	.61–1.42	0.657
Delayed care, non-cost	Yes (ref = no)								.54–1.24	0.213	1.45	.8–2.63	0.102
Health Insurance	Private	1		0.934									
	Public	1.06	.72–1.55										
	None	1.01	.65–1.56										

### Dependent Variables

In the 2007 NHIS supplement, respondents who did not use one or more of the common complementary practices were given ten response options to select from to ascertain their reasons for non-use. The response option “*Never heard of it/Do not know much about it”* (24% of respondents) was selected as the primary dependent variable for analysis. This reason was selected in order to specifically explore the relationship between health knowledge (of complementary health practices) and non-use. For the rest of the article this variable will be referred to as 'lack of knowledge'. A second dependent variable, “*Do not need it”* (43% of respondents), was also selected. For the rest of the article this variable will be referred to as 'lack of need'. These two items were chosen as they were among the most frequently selected, their implied meaning was clearer compared to response options like "*Some other reason*," and they allowed for a parsimonious examination of the interrelated concepts of knowledge and need (particularly, need based on the presence of back pain and the hypothesized search for therapeutic information/knowledge). Associations between these two dependent variables—lack of knowledge and lack of need (as reasons for non-use)—and key independent variables were examined.

### Independent Variables

Twenty-four items were selected as independent variables based on known associations with both use of complementary health practices and back pain status [2–3], [7], [27–31]. These items included: Demographics—eight socio-demographic characteristics (gender, age, race, ethnicity, geographic region, education, income [defined in terms of poverty status], and marital status); Health Status—five variables related to the respondent’s health status (self-reported health status [excellent, very good, good, fair, poor], any functional limitation, hospitalization in the previous 12 months, visits to the emergency room (ER) in the previous 12 months, and back pain); Health Behaviors—five health behaviors and risk factors routinely monitored by the CDC [32] (activity level [inactive, some activity, regular activity], smoking status [current, former, never], alcohol consumption [lifetime abstainer, former drinker, current infrequent drinker, current moderate/heavy drinker], body mass index (BMI) [underweight, healthy weight, overweight, obese], and whether the respondent had received a flu vaccination in the past 12 months and/or ever received the pneumonia vaccine; and Healthcare Access—six variables related to conventional healthcare access and use (usual place for sick care, health insurance coverage, number of visits to a conventional provider in the previous 12 months [0–3, 4–9, 10+], delayed healthcare for reasons of cost, delayed healthcare for reasons other than cost, and ability to pay common ancillary healthcare expenses [prescription medication, mental healthcare, dental care, or prescription eyeglasses]).

Back pain was included as an independent variable in the full sample. It was also used as the variable for stratification in the creation of a second 'back pain only' subsample. Back pain was selected for a subsample analysis as low back pain and other back problems are cited as the most common reasons for use of complementary therapies [2–3]. In addition, low back pain is prevalent in the general population, is clinically and socially costly, and current best practice guidelines for the management of back pain include non-pharmacological complementary health interventions, such as acupuncture, spinal manipulation, massage and yoga [25]. Back pain status was based on the following question in the 2007 NHIS Adult Core—"During the PAST THREE MONTHS, did you have low back pain?”

### Statistical Analyses and Study Limitations

Univariate and multivariate analyses were conducted to examine associations with the two dependent variables—lack of knowledge and lack of need (as reasons for non-use of four common complementary health practices). Logistic regression was used to assess the magnitude and direction of association between each dependent variable and the twenty-four independent variables. To identify variables making substantial contributions to the models, a backwards stepwise methodology was used, with p<0.01 set as the criterion for remaining in the model. An iterative process was then used to examine variables remaining after backwards regression, eliminating those with a relative standard error greater than 100%. Variables remaining at the conclusion of this iterative process were used for the final multiple logistic models.

As the stepwise procedure evaluates variables in the model for collinearity at each step, adding or removing items accordingly, there is no variable collinearity in the final models. Each variable is an independent predictor of the dependent variable being investigated.

All estimates and associated standard errors were generated using SAS [33] and SUDAAN [34] software. SUDAAN was used to account for the complex sample design of the NHIS. All estimates were weighted using the NHIS sample adult record weights. To identify significant relationships with each dependent variable, more conservative 99% confidence intervals were used in both the unadjusted and adjusted analysis because of the enhanced statistical power generated by the large sample size.

A major strength of the present study is that it uses a large sample that is representative of the U.S. civilian non-institutionalized population, thereby increasing generalizability of findings. Limitations include that the NHIS data are self-reported and therefore depend upon the respondent’s memory, willingness and ability to answer the questions accurately. Additionally, these data represent a cross-sectional set of associations and do not allow for trend analysis.

## Results

### Ten Reasons for Non-Use of Four Complementary Health Practices

Table 1a–1c present prevalence statistics on the ten response options that survey respondents could select from as their reasons for non-use. These ten options were presented to respondents who reported non-use of the common complementary health practices. Table 1a presents the full non-user sample (n = 13,128). Of the ten response options provided, four were the most frequently selected: 'No reason', 'Do not need it', 'Never thought about it', and 'Never heard of it' (56%, 43%, 39%, and 27% respectively). Four other options were chosen by less than 5% of participants: 'It costs too much', 'It is not safe to use', 'Medical science has not shown it works', and 'A health care provider told me not to use it'. The rank order of these ten options is essentially the same across the four complementary health practices except for a few differences in yoga.

Table 1b represents non-users who reported low back pain (n = 2,580, 19.0%, SE 0.46). Table 1c represents non-users who did not report low back pain (n = 10,534, 81.0%, SE 0.46). The rank order of the ten response options between these two tables is quite similar. The one consistent difference is that in Table 1b (back pain sample) respondents were less likely to report 'No need'. To identify if this might represent statistically significant differences between the back pain and no back pain groups Z-tests were conducted. The proportion of individuals choosing 'Do not need it' was indeed significantly less (p< 0.001) in those reporting back pain (36.0% SE 1.26) versus those without back pain (44.1%, SE 0.93).

### Characteristics of Non-Users of Complementary Health Practices

S1 Table provides descriptive information on each of the 24 independent variables for the whole sample as well as for those with and without back pain. In the whole sample of non-users the respondents were fairly equally divided by gender and were more likely to be age 25–44 (38%) or 45–64 (31%), non-Hispanic (82%), and white (72%). Many lived in the South (41%), and the majority were not poor (67%). Almost half had at least some college education (48%). Health behaviors were characterized by low levels of physical activity and being more likely to be a current light/infrequent drinker (38%) or lifetime abstainer (31%). Health status was reported as excellent to good with the majority not reporting any functional limitations. Having 3 or more health conditions was common (44%) as were being overweight or obese (62%). Overnight hospitalization in the past 12 months was low (9%), while reported use of emergency room visits was somewhat higher (19%). The overwhelming majority reported a usual place of care (83%), more than half had private health insurance, and few delayed care for cost or other reasons.

### Non-Use Related to Lack of Knowledge and Need, Full Sample

Tables 2 and 3 present logistic regression results examining reasons for non-use of the four complementary practices among all non-users for which there were no missing data in the independent variables (n = 10,589). Only variables that contributed to the models are displayed in the tables. Table 2 shows the relationship between the dependent variable 'lack of knowledge' of the four complementary health practices and the independent variables. Table 3 provides information on the relationship between the dependent variable 'lack of need' and the independent variables.

#### Demographics

The response option 'lack of knowledge', selected as a reason for non-use, shows significant associations with education and poverty (Table 2). Educated respondents, high school or above, were significantly less likely to select lack of knowledge as a reason for non-use of any of the four methods. For example, those who attended college were 58% less likely to select this option as a reason for non-use of acupuncture (OR = 0.42, 99% CI .33-.55, p<.0001). Similarly, those with higher income levels were significantly less likely to select lack of knowledge as a reason for non-use of chiropractic, acupuncture, and yoga. As an example, individuals with higher incomes were 37% less likely to select lack of knowledge related to non-use of chiropractic (OR = 0.63, 99% CI .46-.86, p<.0001).

Conversely, in Table 3, we find individuals with higher educational attainment and higher incomes to be significantly more likely to select the response option 'lack of need' as a reason for non-use of chiropractic, acupuncture and natural products. For example, those who attended college were 22% more likely to select lack of need as a reason for not using chiropractic (OR = 1.22, 99% CI 1.01–1.49, p = .021), and higher income was also associated with non-use of acupuncture (OR = 1.31, 99% CI 1.02–1.68, p = .0008).

In addition, respondents age 18–24 were significantly more likely to report lack of knowledge of acupuncture compared with older individuals. While those in the West were significantly less likely to report lack of knowledge of acupuncture compared with people in the South, (OR = .59, 99% CI .41-.84, p = .002). Respondents in the Northeast were less likely to report lack of need for chiropractic (OR = .66, 99% CI .50-.86, p = .0001).Women were significantly less likely than men to report lack of need for yoga (OR = .69, 99% CI .60-.81, p<.0001). Hispanics were more likely than non-Hispanics to report lack of need for natural products (OR = 1.38, 99% CI 1.13–1.68, p<.0001).

#### Health Status

In Table 2 there was no significant association between the response option 'lack of knowledge' and back pain status. By contrast, in Table 3, those reporting low back pain were significantly less likely to have selected the response option 'lack of need' as a reason for non-use, ranging from 20% for yoga to 35% for chiropractic (e.g., chiropractic OR = .65, 99% CI .53-.78, p<.0001). In addition, those who used emergency room services in the past 12 months were also less likely to report lack of need for all four of the methods (e.g., acupuncture OR = .78, 99% CI .64-.95, p = .001).

#### Health Behaviors

The two health behaviors remaining in the final regression models for both dependent variables—lack of knowledge and lack of need—were physical activity and alcohol consumption. Physical activity was highly statistically significant in relation to both knowledge and need, but in opposite directions. In Table 2, inactive individuals were significantly more likely to select the response option 'lack of knowledge' for all four complementary practices (e.g., acupuncture and regular activity, OR = .67, 99% CI .53-.85, p<.0001). Conversely, in Table 3, active individuals were significantly more likely to select the response option 'lack of need' for three of the complementary approaches, with yoga being the exception.

Related to alcohol use, lifetime abstainers were significantly more likely to report limited knowledge of acupuncture, chiropractic, and yoga compared with respondents at almost all levels of alcohol consumption (e.g., current user of alcohol and chiropractic, OR = .50, 99% CI .37-.67, p<.0001). Conversely, lifetime abstainers were significantly less likely to report lack of need for chiropractic.

#### Healthcare Access

A number of significant associations were found between healthcare access variables and both of the dependent variables. Most consistently, individuals who could not afford ancillary care were more likely to select lack of knowledge and less likely to select lack of need (e.g., lack of need—chiropractic, OR = .82, 99% CI .67–1.0, p = .01).

### Non-Use Related to Lack of Knowledge and Need, Back Pain Sample

A regression was also run using the subset of non-users who had reported low back pain in the previous 3 months, for whom there were no missing data in the independent variables (n = 2,123). Table 4 shows the significant associations of key variables with the response option 'lack of knowledge'. As observed in Table 2, low educational attainment remains significantly associated with non-use of natural products (p<.0001) and yoga (p = .001), and near significance for chiropractic (p = .055). For example, those who had attended college were 50% less likely to select lack of knowledge as a reason for non-use of natural products (OR = 0.50, 99% CI .31-.80, p<.0001). Also similar to the results in Table 2, physical activity was inversely related to selecting 'lack of knowledge' for all four CHPs, more active individuals were less likely to select lack of knowledge.

Table 5 shows the significant associations of key variables with the response option 'lack of need'. In this regression the vast majority of significant associations were lost. Items that remained significant included sex (e.g. yoga and female, OR = .57, 99% CI .39-.85, p = .0003) and alcohol consumption. The remaining significant items were concentrated in healthcare access—having private health insurance, not having a usual place of care, not being able to afford ancillary care. Individuals without a usual place of care or without the ability to afford ancillary care were less likely to select the option ‘lack of need’ as a reason for non-use (e.g. ancillary care and acupuncture, OR = .69, 99% CI .45–1.04, p = .02).

## Discussion

### Non-Use Related to Lack of Knowledge and Need, Full Sample

#### Demographics

As hypothesized, regression results presented in Table 2 show respondents with lower educational attainment and lower income to be significantly more likely to select 'lack of knowledge' as a reason for non-use. In terms of education, those who did not graduate from high school were approximately 29–58% more likely to have selected this reason across the four complementary approaches. These results parallel the health literacy literature for conventional medicine. Findings from the 2003 National Assessment of Adult Literacy (NAAL) have shown low health literacy to be significantly associated with lower educational attainment [35–36]. Relatedly, two studies (n = 581 and n = 351), found a positive association between use of complementary practices and health literacy for all racial/ethnic groups except African Americans [37–38]. Another study looked at CHP use by race in a socioeconomically disadvantaged population, using a regional cohort study dataset. It compared use among white and African American participants (n = 69,214). CHP use was found to be significantly associated with higher educational attainment, higher income, and history of chronic disease for both groups [39].

Similarly, regression results presented in Table 3 show educational attainment to be significantly associated with choosing 'lack of need' as a reason for non-use, but in the inverse direction. Individuals with more education were 8–26% more likely to select 'lack of need' for chiropractic, acupuncture, and natural products. Paradoxically, other national surveys [2–3] have shown higher educational attainment to be associated with greater utilization of complementary health practices, not less. This observed relationship between educational attainment and both the use and non-use of complementary approaches speaks to the role of education as a logical contributor to health knowledge, and consequently, to healthcare decision-making and utilization. One could reasonably expect that greater health knowledge would be related to both the selective use and non-use of complementary health practices based on perceived healthcare/wellness needs and cultural fit.

#### Health Status and Healthcare Access

There were few significant variables in the Health Status or Healthcare Access categories related to selecting 'lack of knowledge' as a reason for non-use. Individuals with functional limitations were less likely to report lack of knowledge of chiropractic, while those who could not afford ancillary care were more likely to report lack of knowledge of yoga and acupuncture. There were also a number of items significantly related to selecting the response option 'lack of need' as a reason for non-use. In particular, respondents who had used emergency room services were significantly less likely to have selected 'lack of need' for any of the four methods. This finding may relate to socioeconomic status and access to care. A Massachusetts study cited challenges with access as a potential contributor to higher ER utilization rates in that state [40]. Other factors associated with higher use of emergency room services have been found to include lower health literacy, poverty, receiving Medicaid coverage, and being elderly (75 and older), disabled, chronically ill, or non-Hispanic black [16, 41].

Respondents were also significantly less likely to select the response option 'lack of need' if they had back pain, lower health status, or were not able to afford ancillary services. It is possible that individuals with lower socioeconomic means, such as many who utilize emergency room services, may be deferring use of complementary therapies for reasons other than lack of need, possibly including limited access to these therapies.

#### Health Behaviors

Physical activity and alcohol use were the two highly significant health behavior variables. Table 2 showed physically inactive respondents to be more likely to choose 'lack of knowledge' as a reason for non-use of all four complementary practices. In contrast, in Table 3 we found physically active respondents to be significantly more likely to choose 'lack of need'. Both of these findings reflect the potential relationships between education, health knowledge/literacy, and informed healthcare decision-making. The CDC's *Health*, *United States*, *2011* presents information on leisure time aerobic and muscle strengthening activity. The report notes a clear relationship between physical activity, education, and income [42]. Individuals with more education, and presumably higher levels of health literacy, are more likely to be physically active. A similar relationship has been observed in relation to use of conventional medicine, where higher levels of physical activity were associated with lower use of conventional healthcare [28]. The implication is that the physically active individual may use exercise as a means to reduce reliance on medical solutions of any variety, complementary or conventional.

Noteworthy in this regard, higher educational attainment was associated with selecting 'lack of need' as a reason for non-use of chiropractic, acupuncture, and natural products, but not for yoga (Table 3). Results of another representative national survey found that individuals who reported using yoga at least once were more likely to be female, urban dwellers, and college educated [43]. Another study of 1,206 symptomatic menopausal women examined use of complementary health practices as a non-pharmacological alternative to hormone replacement therapy. Exercise/yoga was among the most commonly reported practices. In that study the use of alternative methods, such as yoga, was associated with being white, a non-smoker, and physically active [44]. The exception of yoga related to selecting 'lack of need' for CHP methods suggests that the educated respondents may recognize the potential for yoga as part of a physically active lifestyle, although they themselves do not currently use it. Use of yoga in the United States grew significantly between 2002 and 2007 [3]. Given its increasing availability, including integration into conventional gyms and fitness programs, it may be viewed differently than other complementary approaches. This is perhaps most evident in Table 5. Women with back pain were 43% less likely to select 'lack of need' as their reason for not using yoga (OR = .57, 99% CI .39-.85, p = .0003). They did not use yoga, but a lack of need for it was not the reason why.

In terms of alcohol use, lifetime alcohol abstainers were up to 50% more likely to select 'lack of knowledge' as a reason for non-use of all four methods compared with former or current drinkers. They were also less likely to select 'lack of need' as a reason for non-use of chiropractic. Demographic characteristics of lifetime alcohol abstainers may partially explain the relationship between abstinence and non-use of complementary practices. Results of a study using data from the 1988–2006 NHIS Linked Mortality File (n = 41,076) found that current drinkers, versus lifetime infrequent drinkers and abstainers, had higher levels of education, income, lived in the West or Northeast, and were normal weight or overweight [45].

### Non-Use Related to Lack of Knowledge and Need, Back Pain Sample

Low back pain is the most prevalent medical condition related to use of complementary health practices [2–3]. Current best practice guidelines for the management of back pain include several complementary approaches [25]. In addition, individuals with back pain actively seek out information and treatment options for their condition [24]. For these reasons it was hypothesized that survey respondents with back pain would be less likely to report lack of knowledge and lack of need for these four approaches.

In the full sample, looking at the 'back pain status' independent variable, back pain and 'lack of knowledge' were not significantly associated, contrary to expectations. There appeared to be no greater information seeking/health knowledge for those with back pain. However, as might be expected, those reporting back pain were significantly less likely to have chosen 'lack of need' as a reason for non-use.

Considering the role of educational attainment, in the full sample lower educational attainment was found to be very significantly associated with choosing 'lack of knowledge' as a reason for non-use of all four approaches. Individuals with lower levels of education had less health knowledge of the complementary approaches. In the sample comprised solely of those reporting back pain, lower educational attainment remained significantly associated with lack of knowledge for several of the complementary therapies. Thus individuals who were less educated, even with the potentially motivating condition of low back pain, were less likely to have knowledge of these common complementary health practices.

## Conclusion

The findings presented here reflect larger social issues in healthcare in the United States. Use and non-use of complementary health practices is just one aspect of the ongoing complex process of making informed healthcare decisions. Socioeconomic factors, including education and income, contribute significantly to this decision-making calculus. Both education and income have been shown to be important predictors of health disparities [46–47]. Research also reveals that health literacy and communication inequalities may exacerbate such disparities [20], [48–50]. Findings from the current study support these conclusions. Individuals with lower levels of educational attainment were more likely to choose the response option 'lack of knowledge' as a reason for non-use of common complementary health practices. That specific association was observed regardless of back pain status, despite the fact that use of complementary therapies is otherwise highly associated with back pain. It was also found that individuals with lower educational attainment or other socioeconomic indicators were less likely to select the response option 'lack of need' as a reason for non-use. For example, individuals who could not afford ancillary care, who did not have a usual place of care, or who had used emergency room services, were less likely to select lack of need as a reason for non-use.

These results suggest that if individuals with health concerns, such as low back pain, knew about clinically appropriate complementary therapies they might use them. Indeed, a related study examining the relationship between health literacy and clinical outcomes found corroborating evidence. It was reported that in a sample of 310 cognitively intact veterans enrolled in a Veterans Administration primary care clinic, patients with lower health literacy knew less about the various medications they were taking. That difference in understanding, however, did not negatively impact medication adherence or adverse events [51]. Although lack of health knowledge can reduce access to potentially beneficial provider-based and self-care therapies, it does not necessarily preclude utilization if those resources are made appropriately available to patients. Patient-oriented interventions addressing limitations in health knowledge have shown promise, such as tailoring educational interventions based on literacy levels [52–53]. Provider-oriented strategies could include broader implementation of best practice guidelines [54] with low socioeconomic status individuals, including recommendations of complementary therapies. Approaches such as these could help reduce inequities in health knowledge and understanding, and improve access to care for underserved populations.

## Supporting Information

S1 Table Characteristics of non-users of four common complementary health practices. (XLSX)
